# Evaluating the Possible Association between *PD-1 (Rs11568821, Rs2227981, Rs2227982)* and *PD-L1 (Rs4143815, Rs2890658) *Polymorphisms and Susceptibility to Breast Cancer in a Sample of Southeast Iranian Women

**DOI:** 10.31557/APJCP.2020.21.10.3115

**Published:** 2020-10

**Authors:** Shima Karami, Hedieh Sattarifard, Mohammad Kiumarsi, Sahel Sarabandi, Mohsen Taheri, Mohammad Hashemi, Gholamreza Bahari, Saeid Ghavami

**Affiliations:** 1 *Department of Clinical Biochemistry, School of Medicine, Zahedan University of Medical Sciences, Zahedan, Iran. *; 2 *Department of Human Anatomy and Cell Science, Max Rady College of Medicine, Rady Faculty of Health Sciences, University of Manitoba, Winnipeg, MB R3E 3P4, Canada. *; 3 *Genetics of Non- Communicable Disease Research Center, Zahedan University of Medical Sciences, Zahedan, Iran. *; 4 *Children and Adolescent Health Research Center, Resistant Tuberculosis Institute, Zahedan University of Medical Sciences, Zahedan, Iran. *; 5 *Faculty of Medicine, Katowice School of Technology, 40-555 Katowice, Poland. *; 6 *Autophagy Research Center, Faculty of Medicine, Shiraz University of Medical Sciences, Shiraz, Iran. *

**Keywords:** Apoptosis, PD-L1, PD-1, cancer, polymorphism

## Abstract

**Introduction:**

Programmed cell death-1 (*PD-1*) and its ligands (*PD-L1* and PD-L2) play a critical role as a regulator of immune-system cells, including T cell, natural killer T (NKT), monocytes, dendritic cells (DC), and B cells.

**Objective:**

This study aimed to find a possible association between *PD-1* (rs11568821, rs2227981, rs2227982), and *PD-L1* (rs4143815, rs2890658) variants and Breast Cancer (BC) risk in a sample of southeast Iranian women.

**Method:**

The case-control study consisted of 520 individuals, including 260 histologically confirmed BC patients and 260 non-cancer age-matching healthy women as the control group. The Polymerase Chain Reaction-Restriction Fragment Length Polymorphism (PCR-RFLP) and Tetra-Primer Amplification Refractory Mutation System-Polymerase Chain Reaction (T-ARMS-PCR) methods were used for genotyping of *PD-1* (rs11568821, rs2227981, rs2227982), and *PD-L1* (rs4143815, rs2890658) polymorphisms.

**Results and Conclusion:**

Our findings indicated that the *PD-L1* rs4143815 (G/C) variant meaningfully reduced the risk of BC. However, the *PD-L1* rs2890658 variant increased the BC risk in the AC genotype as well as the A allele. Furthermore, we could not find a meaningful association between *PD-1* rs11568821, *PD-1* rs2227981, *PD-1* rs2227982, and BC. Our team examined the possible association between variants and clinicopathological characteristics, including age, size of tumour, lymph node, histology, grade of tumour, estrogen and progesterone receptors status as well as human growth factor receptor 2 (HER2). Our findings demonstrated that *PD-L1* rs4143815, *PD-L1* rs2890658, *PD-1* rs2227982 had a significant association with age. Additionally, we found a significant relation between *PD-1* rs2227982 variant and tumour size. Statistical analyzes of *PD-1* rs2227981 and *PD-1* rs11568821 variants showed a meaningful relation between tumour grade and tumour stage (p=0.006), respectively.

## Introduction

Breast Cancer (BC), the second most frequently diagnosed carcinoma and the primary cause of cancer mortality among women all around the world, is becoming the main concern for public health in all communities (Ferlay et al., 2015). Development of BC begins with the abnormal and rapid division of breast cells , resulting in tumour formation that could invade other breast tissues and spread to other organs (Yu, 2019; Umami et al., 2020) such as brain, lungs, and bones (Eckhardt et al., 2012; Vickers, 2017).

The findings of numerous studies have shown a strong correlation between the susceptibility to BC and factors such as genetic mutation, diet, physical activity, breastfeeding, level of estrogen and progesterone hormones (Jayasekara et al., 2016, Pizot et al., 2016; Anstey et al., 2017; Dall and Britt, 2017; Shiyanbola et al., 2017; Bertoni et al., 2019; McTiernan et al., 2019). However, the leading cause of BC has yet to be identified. Genetic mutation, critical factor to cancer susceptibility, are responsible for permanent alternations in DNA and RNA sequences. Single nucleotide polymorphisms (SNPs) are responsible for phenotype variations in populations (Kitts and Sherry 2002). SNPs’ could be located in various regions of the human genome, such as in promoters, exons, introns, as well as 5′- and 3′ UTRs. Consequently, small changes in these parts might affect the expression of genes and also increase or decrease the vulnerability to certain diseases (Taylor et al., 2001; Bond et al., 2005; Fan et al., 2010; Wu et al., 2014; Schirmer et al., 2016; Hashemi et al., 2020).

Programmed cell death-1 (*PD-1*) is an immune checkpoint key receptor responsible for maintaining self-tolerance and inhibition of uncontrolled inflammation (Francisco et al., 2010; Hashemi et al., 2019; Hashemi et al., 2020). *PD-1* is mainly expressed on the surface of several immune system cells (Inman et al., 2007; Keir et al., 2008; Ahmadzadeh et al., 2009; Hashemi et al., 2019). The human *PD-1* gene is located on chromosome 2q27.3, which consists of two domains, extracellular immunoglobulin V domain and an intracellular domain containing an inhibitory motif (ITIM) and immune receptor tyrosine-based switch motif (ITSM). Programmed cell death ligand 1 and 2 (*PD-L1* and PD-L2) are two main ligands for *PD-1* receptor. ITIM is activated by binding *PD-L1* or PD-L2 ligands to the *PD-1* receptor, which could promote inhibitory signals to decrease the activation of T lymphocytes and cause proliferation (Hashemi et al., 2019). 

*PD-L1*, located at 9p24 chromosome, is one of the transmembrane type 1 glycoproteins. Several studies showed that the upregulation of *PD-L1* is a major cause of cancer immune evasion (Yamazaki et al., 2002; Nguyen and Ohashi, 2015; Salmaninejad et al., 2018; Hashemi et al., 2019). Binding the *PD-L1* ligand with *PD-1* receptor can dephosphorylate several kinases, including ZAP70, AKT, PI3K, and ERK-R, which can inhibit IL-2 production on T cells and cause T cell proliferation and apoptosis (Zheng and Zhou, 2015; Catakovic et al., 2017). The abnormal expression of PD-Ls on the cell lines or tissues of several tumours, including cervical cancer (Karim et al., 2009), gastric carcinoma (Ohigashi et al., 2005), and breast cancer (Ghebeh et al., 2006) have been comprehensively investigated. 

Genetic polymorphism of *PD-L1* and *PD-1* were reported in several malignancies (Haghshenas et al., 2011; Hua et al., 2011; Qiu et al., 2014; Ma et al., 2015; Dougan, 2017; Haghshenas et al., 2017; Juchem et al., 2018; Kuol et al., 2018; Tan et al., 2018; Zhang et al., 2018). Therefore, in the current investigation, our team evaluated the correlation between *PD-1* (rs11568821, rs2227981, rs2227982) and *PD-L1* (rs4143815, rs2890658) and BC susceptibility in a sample of southeast Iranian women. 

## Materials and Methods

*Patients*

Our case-control study was performed on 520 individuals comprising 260 histologically confirmed BC patients, and 260 control healthy individuals within the same age group. The protocol of this study has been designed based on previous investigations (Danesh et al., 2018; Hashemi et al., 2018). The institutional review board approved the current research at Zahedan University of Medical Sciences (IR.ZAUMS.REC.1397.386). Proper consent was obtained from all participants. The genomic DNA samples were extracted using the salting-out method and were collected in separate special tubes containing EDTA (Hashemi et al., 2013).

*Genotyping*

**Figure 1:**
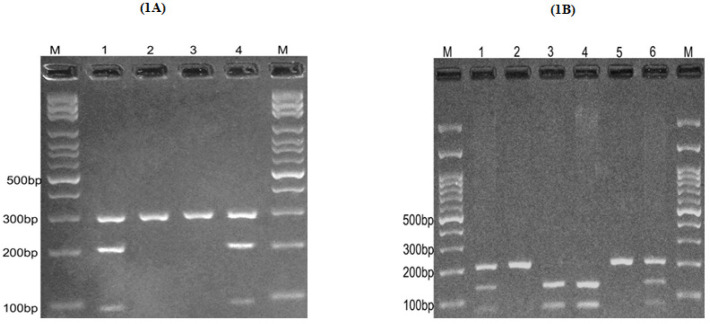
(1A) electrophoresis pattern of the PCR-RFLP method for the detection of *PD-1*rs11568821(G/A) polymorphism. M: DNA marker; Lanes 1 and 4: GA; Lanes 2 and 3: GG (1B): Photograph of electrophoresis pattern of the PCR-RFLP method for detection of *PD-1* rs2227981(C/T) polymorphism. M, DNA marker; Lanes 1 and 6, CT; Lanes 2 and 5, CC; lanes 3 and 4, TT

**Figure 2:**
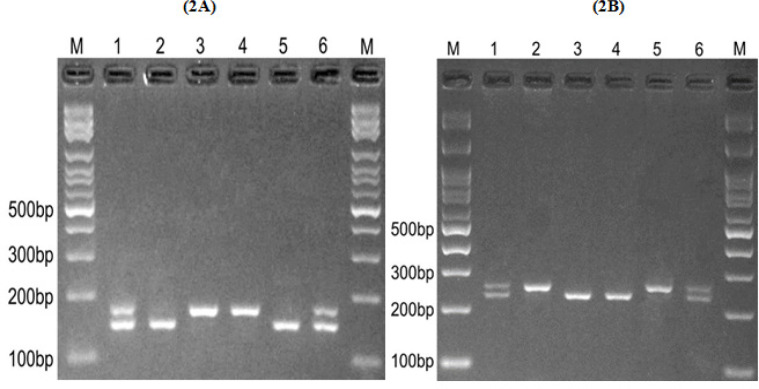
(2A). Electrophoresis pattern of the PCR-RFLP method for the detection of *PD-1*rs2227982(C/T) polymorphism. M: DNA marker; Lanes 1 and 6: CT; Lanes 2 and 5: CC; lanes 3 and 4: TT. (2B) electrophoresis pattern of the PCR-RFLP method for the detection of *PD-L1*rs2890658(C/A) polymorphism. M: DNA marker; Lanes 1 and 6: CA; Lanes 2 and 5: AA; lanes 3 and 4: CC

Polymerase Chain Reaction-Restriction Fragment Length Polymorphism (PCR-RFLP) method was used for genotyping the *PD-L1* rs2890658, *PD-1* rs11568821, *PD-1* rs2227981 and *PD-1* rs2227982 polymorphisms. For identification of the *PD-L1* rs4143815 variant, we used Tetra-Primer Amplification Refractory Mutation System-Polymerase Chain Reaction (T-ARMS-PCR) technique. The procedure used for this investigation, previously designed and testified by Hashemi’s lab, is as follow (Hashemi et al., 2012; Hassanzarei et al., 2017):

1. The volume for assembling the reaction solution in each PCR tube for RFLP-PCR and T-ARMS-PCR methods are accessible in Table 1.

2. Primes sequences used for the detection of *PD-1* and *PD-L1* are presented in Table 2.

3. PCR thermal cycling conditions used for amplification of *PD-1* and* PD-L1* polymorphisms, are listed in Table 3.

4. Corresponding restriction endonucleases used for the digestion of PCR products are mentioned in Table 2.

5. UV transilluminator was used to visualize the fragments digested and separated by agarose gel electrophoresis. Briefly, for *PD-1* rs11568821, the PstI enzyme digested the A allele and produced a 93 base pair (bp) and 197 bp pattern. However, the G allele was undigested (290 bp fragment) Figure 1A. Regarding *PD-1* rs2227981, we used the PvuII restriction enzyme to digest the T allele to a 74 bp and 133 bp pattern. Nevertheless, the C allele remained undigested (207 bp fragment) Figure 1B. For *PD-1* rs2227982, we used the BceAI restriction enzyme to digest the C allele to 29 bp and 145 bp pattern. However, the T allele remained undigested (174 bp fragment) Figure 2A. The *PD-L1* rs2890658 C allele was digested by HaeIII and made a 25 bp and 226 bp pattern, while the A allele stayed undigested (251 bp fragment) Figure 2B. 

**Figure 3:**
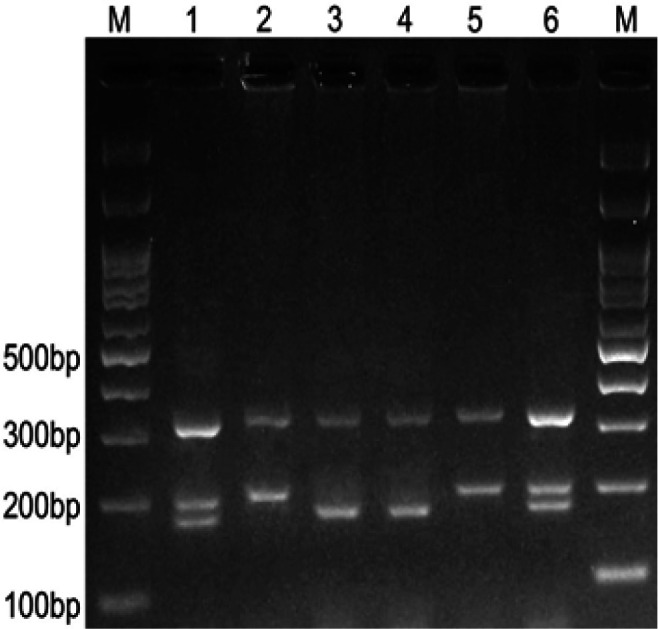
Electrophoresis Pattern of the T-ARMS Method for the Detection of *PD-L1*rs4143815(G/C) Polymorphism. M, DNA marker; Lanes 1 and 6, GC; Lanes 2 and 5, CC; lanes 3 and 4, GG

**Figure 4:**
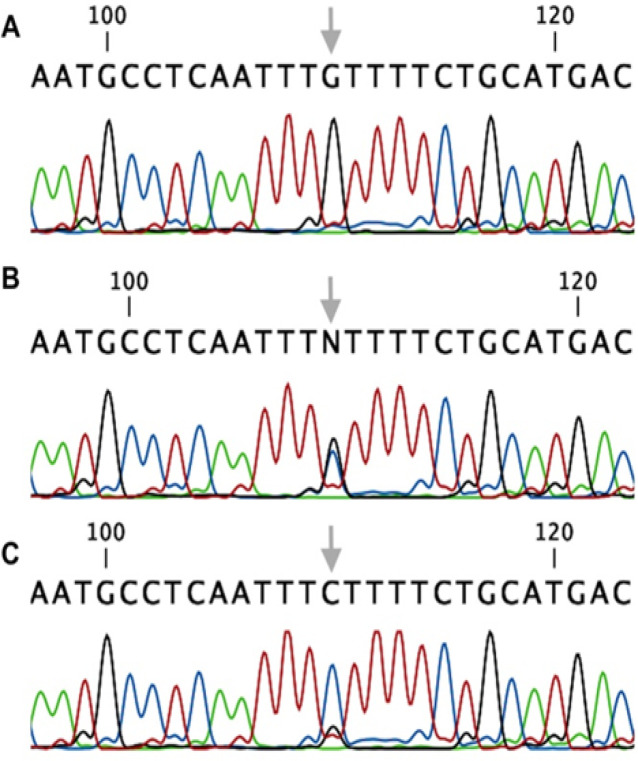
Sequencing of *PD-L1* rs4143815

We used the T-ARMS-PCR method for genotyping the *PD-L1* rs4143815 (Ye, Dhillon et al. 2001, Hashemi, Moazeni-roodi et al. 2012). In this technique, two external primers (forward outer and reverse outer) and two allele-specific internal primers (forward inner and reverse inner) were designed. Three bands were produced; 322 bp for control band, and two 203 bp and 176 bp bands related to C and G allele, respectively Figure 3. Furthermore, we sequenced the *PD-L1* rs4143815 to validate our genotyping and results in Figure 4.

*Statistical analysis*

All of the statistical data was carried out by using computer software Statistical Package for Social Sciences (SPSS) (ver. 24.0). The correlation between *PD-1* (rs11568821, rs2227981, rs2227982) and *PD-L1* (rs4143815, rs2890658) SNPs and BC susceptibility were calculated with Unconditional logistic regression analysis. To estimate the Hardy–Weinberg equilibrium (HWE) among the controls, we used the χ2 test. We considered the p-value less than 0.05 (P<0.05) to be statistically significant.

## Results

The current case-control investigation included 520 participants. The patient group consisted of 260 BC patients with an average age group of 48.09±10.59, and the control group consisted of 260 healthy females with an average age group of 46.26±10.72. There was no significant statistical difference between the age groups (p=0.052). Frequency of alleles and genotyping of *PD-1*(rs11568821, rs2227981, rs2227982) and *PD-L1* (rs4143815, rs2890658) polymorphisms among cases and controls are presented in Table 4.

Our results showed that *PD-L1* rs4143815 (G/C) variant significantly reduced the risk of BC in homozygous (OR=0.52, 95%CI=0.28-0.96, P=0.049, GG vs CC), recessive (OR=0.44, 95%CI=0.26-0.77, P=0.006, GG vs CG+CC) genetic models. Regarding the *PD-L1* rs2890658 C/A polymorphism, our results show that *PD-L1* rs2890658 significantly increased the risk of BC in heterozygous (OR=2.44, 95%CI=1.71-3.46, p<0.0001, CA vs CC), dominant (OR=2.48, 95%CI=1.74-3.51, p<0.0001, CA+AA vs CC), and A allele (OR=1.87, 95%CI=1.41-2.48, p<0.0001, A vs C) genetic models. We could not find a significant correlation between *PD-1* rs11568821, *PD-1* rs2227981, *PD-1* rs2227982 and variants and the risk of BC.

Furthermore, our team examined the possible association between variants and clinicopathological characteristics, including age, size of the tumour, lymph node, histology, the grade of tumour, estrogen and progesterone receptors status as well as human growth factor receptor 2 (HER2) Table 5. Our findings demonstrated that *PD-L1* rs4143815, *PD-L1* rs2890658, and *PD-1* rs2227982 had a significant correlation with age (p=0.005, p=0.046, p<0.001). Additionally, we found a significant correlation between *PD-1* rs2227982 variant and tumour size (p=0.049). Statistical analyzes of *PD-1* rs2227981 and *PD-1* rs11568821 variants showed the meaningful relation between tumor grade (p=0.049) and tumor stage (p=0.006).

**Table 1:** The Volumes for Assembling the Reaction Solution in Each PCR tube for the Detection of *PD-1* and *PD-L1 *Polymorphisms

Polymorphisms	Reverse Inner Primer	Forward Inner Primer	Forward Outer Primer	Reverse Outer Primer	2X Taq master mix	H_2_O	DNA
*PD-L1* Rs4143815 Tetra-ARMS	1 µl	1 µl	1 µl	1 µl	8 µl	6 µl	1 µl
*PD-L1* Rs2890658 RFLP	1 µl	1 µl	-	-	8 µl	6 µl	1 µl
*PD-1* Rs11568821 RFLP	1 µl	1 µl	-	-	8 µl	6 µl	1 µl
*PD-1* Rs2227981 RFLP	1 µl	1 µl	-	-	8 µl	6 µl	1 µl
*PD-1* Rs2227982 RFLP	1 µl	1 µl	-	-	8 µl	6 µl	1 µl

**Table 2:** The Pprimers Used for Detection of *PD-1* and *PD-L1* Polymorphism

Polymorphisms	Primer sequence (5`->3`)	Restriction Enzyme	Fragment (bp)
*PD-L1* rs4143815	FO: CTGTGACAGGGAGAAAGGATACTTCTG RO: AGCAAGTTTAGTTTGGCGACAAAATTGT FI: TTTGCCTCCACTCAATGCCTCAATATC RI: AACACTGAGACTCTCAGTCATGCAGAATAC	--	Allele G=176 bp Allele C=203 bp Control= 322 bp
*PD-L1* rs2890658	F: GCAAGAGGAAGTGAAATAATCAAG R: GATACCTGTGTTAAAATGGGAACAG	HaeIII	Allele A=251 bp Allele C= 226+25 bp
*PD-1* rs11568821	F: CTCACATTCTATTATAGCCAGGACC R: TAAGATAAGAAATGACCAAGCCCAC	PstI	Allele G=290 bp Allele A=197+93 bp
*PD-1* rs2227981	F: TGAGCAGACGGAGTATGCC R: CTGAGGAAATGCGCTGACC	PvuII	Allele C=207bp Allele T= 133+74 bp
*PD-1* rs2227982	F: TGACTCCCTCTCCCTTTCTCCTC R: GCCCATTCCGCTAGGAAAGA	BceAI	Allele T=174 bp Allele C=145+29 bp

**Table 3:** PCR Thermal Cycling Conditions for Amplification of *PD-1* and *PD-L* Polymorphisms

polymorphism	Denaturation		Annealing		Extension		Cycles
	Time	Temp	Time	Temp	Time	temp	
*PD-L1* rs4143815	30s	95^0^C	30s	58^0^C	30s	72^0^C	30
*PD-L1* rs2890658	30s	95^0^C	30s	59^0^C	30s	72^0^C	30
*PD-1* rs11568821	30s	95^0^C	30s	64^0^C	30s	72^0^C	30
*PD-1* rs2227981	30s	95^0^C	30s	61^0^C	30s	72^0^C	30
*PD-1* rs2227982	30s	95^0^C	30s	62^0^C	30s	72^0^C	30

**Table 4:** The Association of *PD-1* and *PD-L1 *Polymorphisms and Breast Cancer Risk

*PD-L1 *Polymorphisms	Case	Control	OR (95%CI)
	n (%)	n (%)	
*PD-L1 rs4143815*			
Codominant			
CC	79 (30.4)	84 (32.3)	1
CG	161 (61.9)	135 (51.9)	1.27 (0.87-1.86)
GG	20 (7.7)	41 (15.8)	0.52 (0.28-0.96)
Dominant			
CC	79 (30.4)	84 (32.3)	1
CG+GG	181(69.6)	176 (67.7)	1.09 (0.75-1.60)
Recessive			
CG+CC	240 (92.3)	219 (84.2)	1
GG	20 (7.7)	41 (15.8)	0.44 (0.26-0.77)
Allele			
C	319 (61.35)	303 (58.27)	1
G	201 (38.65)	217 (41.73)	0.88 (0.68-1.13)
*PD-L1 rs2890658*			
Codominant			
CC	101 (38.9)	159 (61.1)	1
CA	155 (59.6)	100 (38.5)	2.44 (1.71-3.46)
AA	4 (1.5)	1 (0.4)	6.30 (0.69-57.14)
Dominant			
CC	101 (38.9)	159 (61.1)	1
CA+AA	159 (61.1)	101 (38.9)	2.48 (1.74-3.51)
Recessive			
CC+CA	256 (98.5)	259 (99.6)	1
AA	4 (1.5)	1 (0.4)	4.05 (0.45-36.45)
Allele			
C	357 (68.7)	418 (80.3)	1
A	163 (31.3)	102 (19.7)	1.87 (1.41-2.48)
*PD-1 rs11568821*			
Codominant			
GG	234 (90.0)	245 (94.2)	1
GA	26 (10.0)	15 (5.8)	1.81 (0.95-3.39)
AA	0	0	
Allele			
G	494 (95.0)	505 (97.1)	1
A	26 (5.0)	15 (2.9)	1.77 (0.95-3.35)
*PD-1 rs2227981*			
Codominant			
CC	113 (43.5)	130 (50.0)	1
CT	139 (53.4)	125 (48.1)	1.28 (0.90-1.82)
TT	8 (3.1)	5 (1.9)	1.84 (0.53-5.78)
PD-L1 Polymorphisms	Case	Control	OR (95%CI)
	n (%)	n (%)	
Dominant			
CC	113 (43.5)	130 (50.0)	1
CT+TT	147 (56.5)	130 (50.0)	1.30 (0.93-1.83)
Recessive			
CC+CT	252 (96.9)	255 (98.1)	1
TT	8 (3.1)	5 (1.9)	1.62 (0.51-4.44)
Allele			
C	365 (70.2)	385 (74.0)	1
T	155 (29.8)	135 (26.0)	1.21 (0.93-1.59)
PD-1 rs2227982			
Codominant			
CC	211(81.2)	200 (76.9)	1
CT	47 (18.1)	56 (21.5)	0.79 (0.52-1.23)
TT	2 (0.7)	4 (1.6)	0.47 (0.09-2.05)
Dominant			
CC	211 (81.2)	200 (76.9)	1
CT+TT	49 (18.8)	60 (23.1)	0.77 (0.50-1.18)
Recessive			
CC+CT	258 (99.3)	256 (98.4)	1
TT	2 (0.7)	4 (1.6)	0.50 (0.09-2.15)
Allel			
C	469 (90.2)	456 (87.7)	1
T	51 (9.8)	64 (12.3)	0.77 (0.53-1.15)

**Table 5:** Association of *PD-1* and *PD-L1 *Polymorphisms with ClinicoPathological Characteristics of Breast Cancer (BC) Patients

Characteristic of patients	PD-L1 rs4143815			P value	PD-L1 rs2890658			P value	PD-1 rs11568821			P value	PD-1 rs2227981			P value	PD-1 rs2227982			P value	P value
	CC	CG	GG		CC	CA	AA		GG	GA	AA		CC	CT	TT		CC	CT	TT		
Age, years				0.005				0.046				0.3				0.956				<0.001	0.368
≤50	55	82	15		58	94	0		134	18	0		67	80	5		112	40	0		
>50	24	77	4		42	59	4		97	8	0		45	57	3		99	5	1		
Tumor size, cm				0.07				0.351				1				0.558				0.049	0.289
≤2	12	38	1		18	33	0		47	4	0		26	23	2		45	5	1		
>2	54	105	18		74	99	4		160	17	0		76	95	6		142	35	0		
Histology				0.156				0.766				0.031				0.288				0.223	0.02
Ductal carcinoma	67	131	14		84	125	3		187	25	0		88	116	8		172	39	1		
Others	7	22	5		13	21	0		34	0	0		18	16	6		29	4	1		
Lymph node metastasis				0.353				0.27				1				0.635				0.158	0.123
No	17	37	7		23	38	0		55	6	0		23	36	2		46	14	1		
Yes	42	91	8		64	74	3		127	14	0		63	73	5		118	23	0		
Grade				0.909				0.858				0.951				0.049				0.543	0.007
I	10	20	2		14	17	1		29	3	0		18	13	1		28	4	0		
II	33	67	7		40	65	2		96	11	0		46	54	7		85	20	2		
II+IV	23	58	8		38	50	1		79	10	0		35	54	0		72	17	0		
Stage				0.659				0.413				0.006				0.72				0.623	0.07
I	5	7	2		4	10	0		13	1	0		6	8	0		13	1	0		
II	28	74	8		45	63	2		106	4	0		43	62	5		87	22	1		
III	24	38	4		29	35	2		53	13	0		32	32	2		56	10	0		
IV	11	19	2		8	24	0		28	4	0		13	19	0		26	5	1		
Estrogen receptor status				0.293				0.509				0.257				0.857				0.679	0.9399
Positive	54	93	13		67	90	3		147	13	0		70	84	6		134	25	1		
Negative	20	56	7		29	53	1		72	11	0		37	44	2		66	16	1		
Progesterone receptor status				0.777				0.895				0.371				0.547				0.909	0.852
Positive	48	91	14		61	89	3		140	13	0		64	83	6		126	26	1		
Negative	26	55	6		35	51	1		76	11	0		42	43	2		72	14	1		
HER2 status				0.581				0.61				0.166				0.477				0.327	0.953
Positive	21	49	8		28	48	2		67	11	0		38	37	3		62	16	0		
Negative	53	100	12		67	96	2		152	13	0		68	92	5		139	24	2		

## Discussion

Our current study aimed to examine the possible association between *PD-1* (rs11568821, rs2227981, rs2227982), *PD-L1* (rs4143815, rs2890658) polymorphisms and susceptibility to BC in a sample of southeast Iranian women. Recent investigations showed that *PD-1* and its ligand (*PD-L1*) play a critical role in regulation of immune system cells’ function (Inman et al., 2007; Keir et al., 2008). Several studies showed that there is a meaningful association between the abnormal expression of *PD-1*/PD-Ls and susceptibility to BC (Haghshenas et al., 2011, Hua et al., 2011; Ren et al., 2016). Thus, we considered the *PD-1*/*PD-L1* pathway as a strong potential candidate for susceptibility to BC in a sample of southeast Iranian women. 

Our result proposed that the GG genotype of the *PD-L1* rs4143815 variant significantly decreased the risk of BC in our study. We also found that *PD-L1* rs4143815 had a significant correlation with age (Table 5). Currently, there is still controversy about the direct function of *PD-L1* rs4143815 in cancers. Several studies suggested that abnormal expression of *PD-L1* rs4143815 increased the susceptibility risk to gastric cancer (Wang et al., 2013), ovarian cancer (Tan et al., 2018), and hepatocellular (HCC) carcinoma (Xie et al., 2018) in the Chinese population. However, there was no relation between *PD-L1* rs4143815 and susceptibility or protection to esophageal squamous cell carcinoma (Zhou et al., 2016) and colorectal cancer (Catalano et al., 2018).

Regarding the *PD-L1* rs2890658 C/A polymorphism, our results showed that the AC genotype and A allele of *PD-L1* rs2890658 significantly increased the risk of BC. Similarly, Chen et al., (2014) showed that AC genotype and A allele increased the risk of non-small cell lung cancer (NSCLC) susceptibility in a Chinese population. Furthermore, *PD-L1* rs2890658 was also recognized as a risk factor in NSCLC in two other studies of the Chinese population (Cheng et al., 2015; Ma et al., 2015). However, there is still controversy about the primary role of *PD-L1* rs2890658 in cancer susceptibility. Xie et al., (2018) found that *PD-L1* rs2890658 was not associated with HCC in a Chinese population. We also found that *PD-L1* rs2890658 had a significant relationship with age (Table 5).

*PD-1* rs11568821 (*PD-1*.3) is located on intron 4 of the *PD-1* human gene. A “G” to “A” alteration might affect the runt-related transcription factor 1 (RUNX1) binding site (Prokunina et al., 2002). Our findings showed no meaningful differences in genotype and allele frequencies of *PD-1* rs11568821 variant and BC risk. Therefore, we concluded that RUNX1 activity was not correlated with BC risk in our study. Similar to our result, Haghshenas (2011) reported that the *PD-1* rs11568821 variant was not associated with BC and thyroid cancer in a sample of south Iranian females (Haghshenas et al., 2011). Furthermore, numerous investigations showed that *PD-1* rs11568821 was not associated with NSCLC (Ma et al., 2015), Leukemia (Ramzi et al., 2018), colorectal cancer (Yousefi et al., 2013), Benign Brain Tumors (Namavar et al., 2017) and hepatocellular carcinoma (Bayram et al., 2012). We also found that there is a significant association between *PD-1* rs11568821 and the stage of tumour in BC patients (Table 5).

In the current research, we could not find any correlation between *PD-1* rs2227981 and BC. Similarly, numerous studies all around the world could not confirm a significant association between *PD-1* rs2227981 and susceptibility or protection to BC (Haghshenas et al., 2011), colorectal cancer (Savabkar et al., 2013), NSCLC (Ma et al., 2015) and epithelial ovarian cancer (Li et al., 2016). However, *PD-1* rs2227981 variant was recognized as a risk factor in several cancers including, cervical cancer (Li et al., 2016), BC (Hua et al., 2011), gastric cancer (Savabkar et al., 2013) and thyroid cancer (Haghshenas et al., 2017). Our findings also showed that *PD-1* rs11568821 is significantly associated with tumour grade (Table 5).

In the current study, we also confirmed that there was not any significant correlation between *PD-1* rs2227982 and BC. Similarly, several investigations were not able to find any association between *PD-1* rs2227982 variant and susceptibility or protection to BC (Hua et al., 2011), esophageal cancer (ESCC) (Qiu et al., 2014) and non-small cell lung cancer (NSCLC) (Ma et al., 2015). Despite our results, the *PD-1* rs2227982 variant was recognized as a protective factor in esophagogastric junction adenocarcinoma (Tang et al., 2017). However, the result of many studies revealed that *PD-1* rs2227982 variant was a risk factor in several cancers including, leukemia(Ramzi et al., 2018), gastric cardia adenocarcinoma (Tang et al., 2017), esophageal squamous cell carcinoma (Zhou et al., 2016) and ovarian cancer (Tan et al., 2018). Our findings also demonstrated that *PD-1* rs2227982 was significantly associated with age in the patient group (Table 5).

In conclusion, our current study suggested that two functional polymorphisms *PD-L1* rs4143815 and *PD-L1* rs2890658 were associated with BC protection and risk in a sample of southeast Iranian women. The previous investigations have shown that *PD-1* and *PD-L1*/PD-L2 belong to the family of immune checkpoint proteins which could induce proliferation and apoptosis in T Cells of cancer patients and causes cancer development (Li et al., 2016; Sacher and Gandhi, 2016, Hashemi et al., 2019). Thus, genetic variation in *PD-L1* rs4143815 and *PD-L1* rs2890658 could be a possible prognostic marker for the prediction of BC susceptibility and development. Additionally, we could not find a meaningful association between *PD-1* rs11568821, *PD-1* rs2227981, *PD-1* rs2227982 variants and BC risk or protection. Inconsistency in the results of several investigations might be associated with different genetic backgrounds, environmental factors, and the sample size of the study. Further investigations and larger sample sizes are needed to clarify the primary function of *PD-1* (rs11568821, rs2227981, rs2227982), *PD-L1* (rs4143815, rs2890658) polymorphisms and BC susceptibility.
